# The effect on growth of *Chlamydomonas reinhardtii* of flue gas from a power plant based on waste combustion

**DOI:** 10.1186/s13568-014-0049-4

**Published:** 2014-06-18

**Authors:** Leiv M Mortensen, Hans R Gislerød

**Affiliations:** 1 Department of Plant Science, The University of Life Sciences, Ås NO-1432, Norway

**Keywords:** Carbon dioxide concentration, Chlamydomonas reinhardtii, Flue gas, Photosynthetic active radiation

## Abstract

Flue gases from a power plant based on waste combustion were tested as a carbon dioxide (CO_2_) source for growing *Chlamydomonas reinhardtii*. To achieve recognition as an environmentally friendly hydrogen production method, waste gases should be used to grow this hydrogen-producing microalgae. The algae were grown in undiluted flue gas containing 11.4±0.2% CO_2_ by volume, in diluted flue gas containing 6.7±0.1% or 2.5±0.0% CO_2_, and in pure liquid CO_2_ at a concentration of 2.7±0.2%. The NO_x_ concentration was 45±16 mg m^-3^, the SO_2_ concentration was 36±19 mg m^-3^, the HCl concentration 4.1±1.0 mg m^-3^ and the O_2_ concentration 7.9±0.2% in the undiluted flue gas. Undiluted flue gas reduced the dry weight production by around 20-25% when grown at a photon flux density (PFD) of 300 μmol m^-2^ s^-1^ artificial light and at 24 or 33°C, compared with the other treatments. A less negative effect was found at the highest flue gas concentration when the algae were grown at 75 μmol m^-2^ s^-1^ PFD. Growing the algae outdoors at a day length of 12.5 h and a temperature of around 24°C, the dry weight production was higher (about 15%) in the 2.6% CO_2_ flue gas treatment compared with all other treatments. Reducing the light level by 30% through shading did not affect the dry weight production. Calculated on aerial basis the productivity reached approximately 70 g m^-2^ day^-1^ in the 300 μmol m^-2^ s^-1^ PFD treatment (corresponding to 25 mol m^-2^ day^-1^) and approximately 17 g m^-2^ day^-1^ in the 75μmol m^-2^ s^-1^ PFD treatment (corresponding to 6.5 mol m^-2^ day^-1^). The outdoor production reached around 14 g m^-2^ day^-1^. It was concluded that the negative effect of the undiluted flue gas was attributable to the high CO_2_ concentration and not to the other pollutants.

## Introduction

The single-celled green alga *Chlamydomonas reinhardtii* is known to produce hydrogen when starved of sulphur under anaerobic conditions (Skjånes et al. [2007]; Nguyen et al. [2011]; Geier et al. [2012]). At present, conventional hydrogen production is energy-intensive, and more environmentally friendly production based on biological processes is therefore of great interest (Jo et al. [2006]). Today, the atmospheric CO_2_ concentration of about 400 μmol mol^-1^ strongly limits the algal growth, and additional CO_2_ gas has to be supplied throughout the production phase (Geier et al. [2012]). Waste CO_2_ from industrial flue gases should be used in order to make the production environmentally friendly. This will also contribute to reducing CO_2_ emissions that are important to the environment (IPCC [2013]). Several studies have been carried out on the effect of flue gases on the growth of microalgae (Douskova et al. [2009]; Kastanek et al. [2010]; Borkenstein et al. [2011]). *Chlamydomonas reinhardtii* seems to have been little studied, however (see review by van den Hende et al. [2012]). Flue gases contain pollutants such as NO_x_ and SO_2_ that can reach harmful levels depending on the species (van den Hende et al. [2012]). However, few studies have devoted attention to whether the harmful effects depend on environmental factors such as irradiance level and temperature. In tomato plants, it is known that susceptibility to NO_x_ is much higher in low-light as opposed to high-light conditions (Mortensen [1986]). For microalgae, and particularly for *C. reinhardtii,* little is known about the modifying effects of climate factors. Therefore, in this work the effect of flue gas was studied on *C. reinhardtii* at different levels of artificial light and in outdoor conditions with and without shade, as well as at two temperature levels.

## Material and methods

*Chlamydomonas reinhardtii* strain SAG 34.89 from SAG (Göttingen, Germany) obtained from the NIVA culture collection, Norway, was used in the experiments. The algae were stored on Petri dishes covered with TAP medium 1.5% agar (Gorman and Levine [1965]). The algae were grown in the high-salt Sueoka medium (Sueoka [1960]). Sodium bicarbonate was used in the medium to buffer the culture at 10 mM. The microalgae were grown in 1.0 l clear plastic bottles (80 mm inner and 82 mm outer diameter) filled with 0.85 l of growing medium (filled up to 17 cm). Tubes with these dimensions have a volume of approximately 60 l per m^2^ surface area when placed closely together, as the bottles were in the present experiments. The light was supplied by cool white fluorescence tubes (Osram L58W/840) 24 h day^-1^ placed about 10 cm in front of the row of bottles. The photon flux density (PFD) of the artificial light was measured by a LI-COR Model Li-250 instrument with quantum sensor (400-700 nm). The light was supplied from one side and was measured at the surface of the bottles. However, inside the culture the light level strongly decreased from the light exposed side to the opposite side of the bottles, as well as with increasing cell concentration during growth. Typically, the light level decreased by about 70% through the 8.0 cm diameter bottle at start of the experiment and by more than 99.9% at the end of the experiment, due to the increase in the algae concentration.

Two experiments were carried out indoor with artificial light, while a third experiment was carried out outdoor in daylight. The daylight was measured by a Delta-T Devices PAR sensor (cosine corrected within ±5% up to 70° incidence). The temperature was controlled by placing the bottles with the microalgae culture in water baths controlled by aquarium heaters. A circulation pump ensured a homogenous temperature in the water baths. The temperature was measured by cupper-constantan thermocouples. The CO_2_ concentration was measured by a Vaisala CO_2_ transmitter (Type GMT221, range 0-5%). The CO_2_ concentration as well as the temperatures and the daylight PAR were recorded as hourly means by a Campbell CR10X logger with an AM25T thermocouple multiplexer. In addition a Vaisala GMP instrument was used to measure the CO_2_ concentrations between 0 and 20%, and the measurements were recorded as hourly means.

### The flue gas

**Figure 1:**
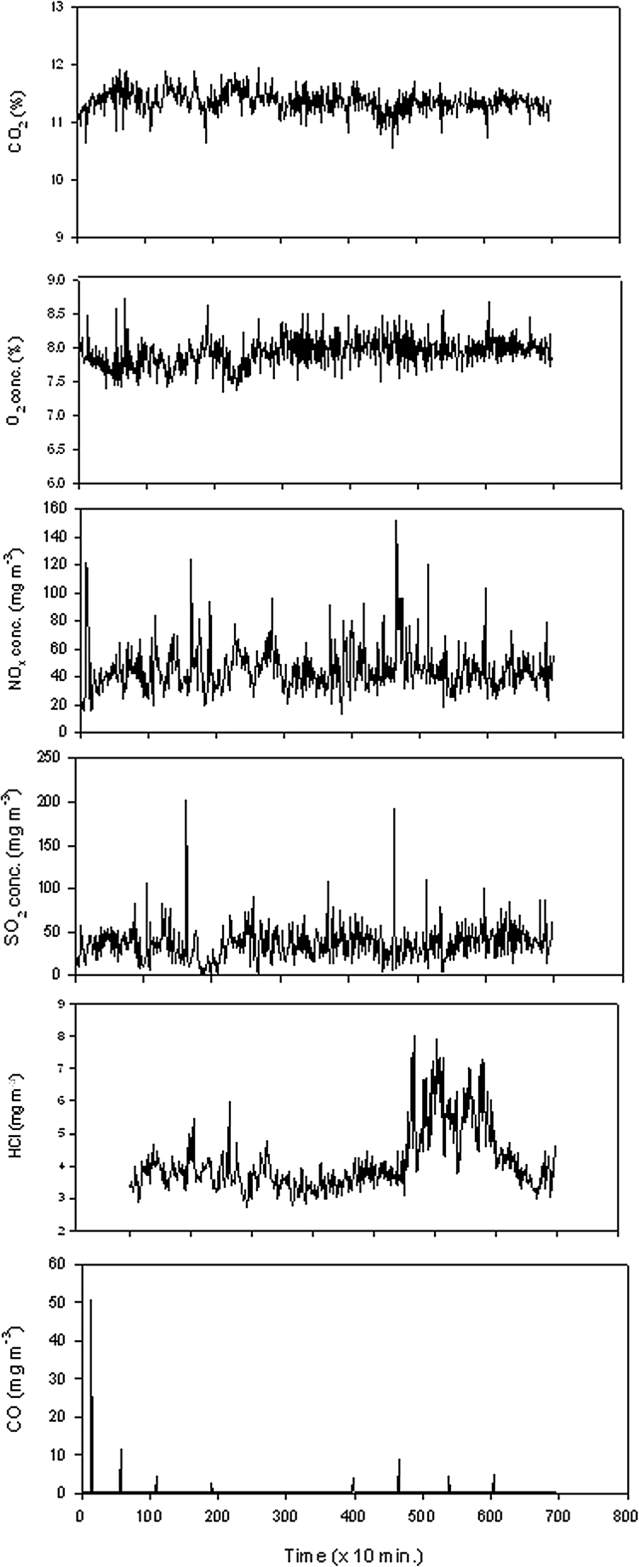
The concentration of different gases in undiluted flue gas.

The flue gas was provided by ‘Borregaard Waste to Energy’ located in Sarpsborg, Norway (www.hafslund.no). This modern fuel-flexible energy recovery plant burns approximately 80,000 tonnes of waste-based fuel and produces approximately 230 GWh per year. It has a high environmental standard. The CO_2_, O_2_, NO_x_, NO, NO_2_, SO_2_, HCl, CO and TOC concentrations in the flue gas were measured at 10-minute intervals by an ABB Advance Cemas FTIR NT continuous monitoring system with extra modules for O_2_ and TOC measurements (Figure 1, Table 1). NO constituted the main part of the NO_x_, while NO_2_ contributed only 3.4±1.4% of the total NO+NO_2_ (data not presented). The mean O_2_ concentration in the flue gas was 7.9±0.2%. In addition, license measurements on a series of heavy metals and dioxins in the flue gas were performed 2-4 times per year since the start of the power plant in 2010 (Table 1).

**Table 1:** Mean concentrations (±SD) of different pollutants as measured in the different flue gas concentrations

	**CO**_**2**_**conc. (%)**		
	**11.37±0.19**	**6.71±0.11**	**2.50±0.04**
NO_x_ (mg m^-3^)	45.0±15.8	26.6±9.3	9.9±3.5
SO_2_ (mg m^-3^)	36.1±19.0	21.3±11.2	7.9±4.2
HCl (mg m^-3^)	4.11±0.95	2.43±0.56	0.90±0.2
CO (mg m^-3^)	0.45±2.00	0.27±1.18	0.10±0.4
TOC (mg m^-3^)	0.714±0.436	0.421±0.257	0.157±0.096
*Hg (μg m^-3^)	0.28±0.35	0.17±0.21	0.062±0.046
*HF (mg m^-3^)	0.063±0.020	0.037±0.012	0.014±0.004
*Dioxins (ng m^-3^)	0.00151±0.00134	0.00089±0.00079	0.00033±00029
*As+Co+Cr+Cu+Mn	0.0211±0.0545	0.0124±0.0322	0.0046±0.0120
+Ni+Pb+Sb+V (mg m^-3^)			

The concentrations were measured continuously in the undiluted flue gas (11.37%) and the concentrations in the diluted flue gases were reduced to the same extent as the CO_2_ concentration. *These concentrations were measured 2-4 times per year in the period 2010-2013 (n=10, ±SD).

Flue gas from the chimney was sucked by pumps through two 100 l plastic tubs connected in series for condensation of water vapour. The microalgae were grown in undiluted flue gas (11.4% CO_2_) or mixed with fresh air in a constant ratio using air pumps (Resun ACO-008A) to yield 6.7% and 2.5% CO_2_, respectively (Figure 1, Table 1). One CO_2_ concentration (2.66±0.16%) was established by mixing pure CO_2_ (food quality) from bottles with fresh air. The CO_2_ gas flow was determined by a capillary with a defined resistance, while the gas pressure was defined by the height of a water column. In this way, a very accurate CO_2_ flow could be added to a constant rate of fresh air supplied by air pumps (Resun ACO-001, ACO-004).

The different gas mixtures were bubbled through plastic tubes with 0.3 cm inner diameter to the bottom of the bottles at a rate of approximately 100 l h^-1^. All treatments in all experiments included three parallel bottles containing 0.85 l of culture. Three independent experiments (including a total of 60 bottles) were carried out during the same time period, all of which started with the same algae concentration of 0.20 g dry weight per litre culture. This concentration was established by adding algae from a start culture. Two of the experiments were conducted indoor with artificial lighting while the third was conducted outdoor in daylight.

### Dissolved CO_2_ in the growth medium

**Figure 2:**
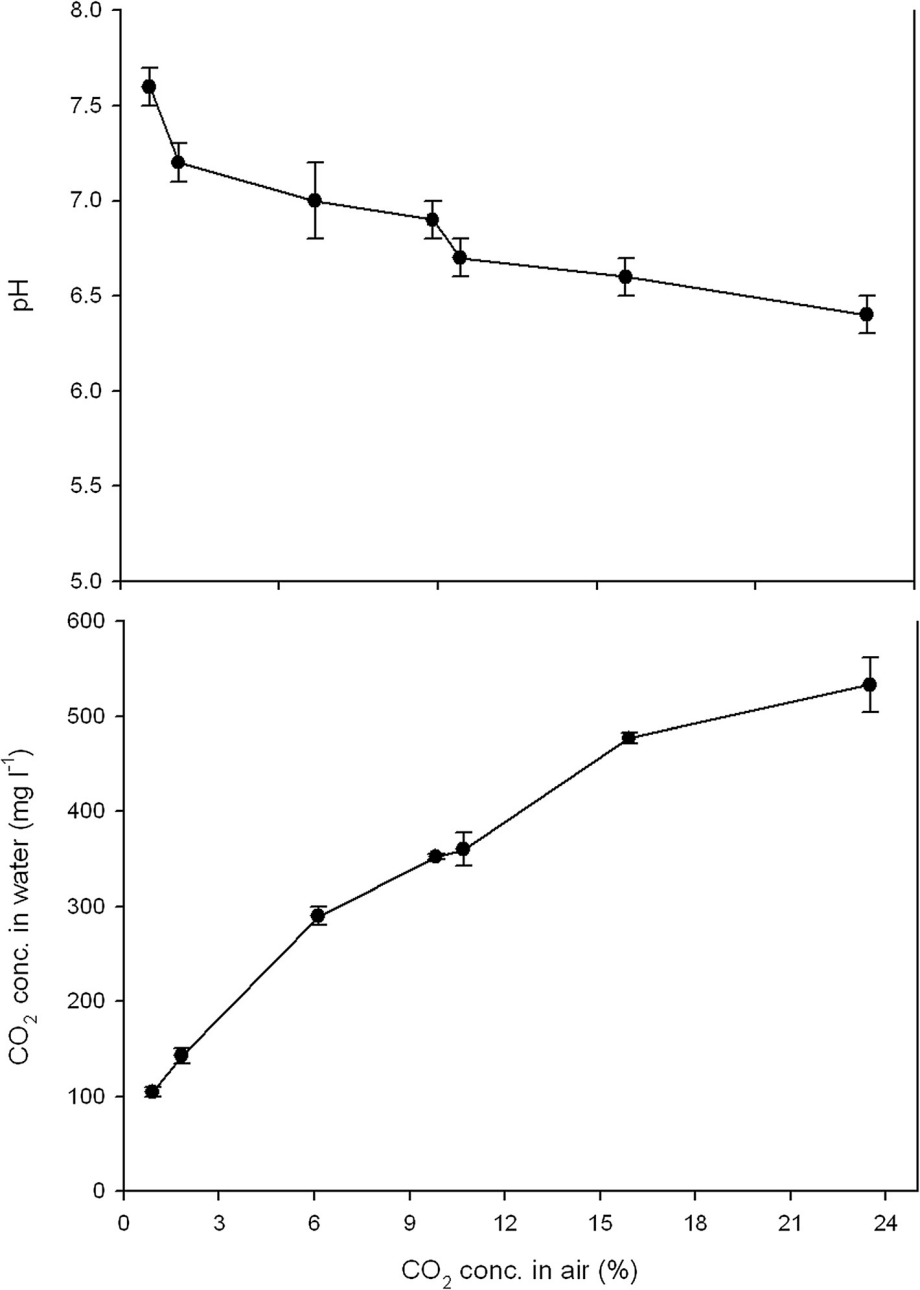
**The concentration of CO**_**2**_**dissolved in the culture and pH of the growth medium as influenced by the CO**_**2**_**concentration in the air bubbled through the bottles (means, ±SE).**

For algal growth, the concentration of dissolved CO_2_ in the nutrient medium is important and not the concentration of CO_2_ in the air bubbled into the culture, although a close relationship should be expected. In order to document this relationship a test with different concentrations of pure CO_2_ mixed with air were bubbled through the bottles filled with nutrient medium. The concentration of dissolved CO_2_ was measured using hand-held titration cells for titrimetric analysis (CHEMetrics Inc., USA, www.chemetric.com). The results showed that a progressive increase in the dissolved CO_2_ concentration from about 100 to about 500 mg l^-1^ with increasing CO_2_ concentration from about 1% up to about 20% (Figure 2). Parallel to this increase the pH decreased from 7.6 to about 6.5. The measurements were done at 23°C. Dissolved CO_2_ as measured at 7.0% CO_2_ in the air was 311±12, 297±12 and 297±12 mg l^-1^ (n=3, ±SE) at 23, 28 and 33°C, respectively.

### The experiments

#### Experiment 1

The microalgae were grown at the three flue gas concentrations and one concentration with pure CO_2_ from bottles (Figure 1, Table 1). Two photon flux densities (PFD) were continuously applied, 75 and 300 μmol m^-2^ s^-1^, corresponding to 6.5 and 25.9 mol m^-2^ day^-1^ PAR, respectively. Two rows of twelve bottles with algae culture were placed closely adjacent to each other in a water bath. One row along one side of the water bath was exposed to 300 μmol m^-2^ s^-1^ PFD, and the other row along the opposite side was exposed to 75 μmol m^-2^s^-1^ PFD. A black sheet across the water bath eliminated any light pollution between the two light treatments. The water bath was made of transparent plexiglass, and one and four fluorescent tubes placed 10-15 cm from the bottles (outside the water bath) produced the low and high PFD, respectively. The temperature was 33±2°C. The dry weight (mg l^-1^ culture), pH and O_2_ concentration in the culture were measured after three and five days, and the production per m^2^ and day was calculated using the vertical projected area of the bottles.

#### Experiment 2

The same flue gas and pure CO_2_ gas treatments were applied in this experiment as in Experiment 1. In this experiment a PFD of 300 μmol m^-2^ s^-1^ given continuously was used. The temperature was 19±2°C during the first day, and was thereafter increased to 24±2°C. The temperature was controlled as in Experiment 1. Twelve bottles were included in the experiment, and the dry weight concentration and pH were measured four and five days after the start.

#### Experiment 3

**Figure 3:**
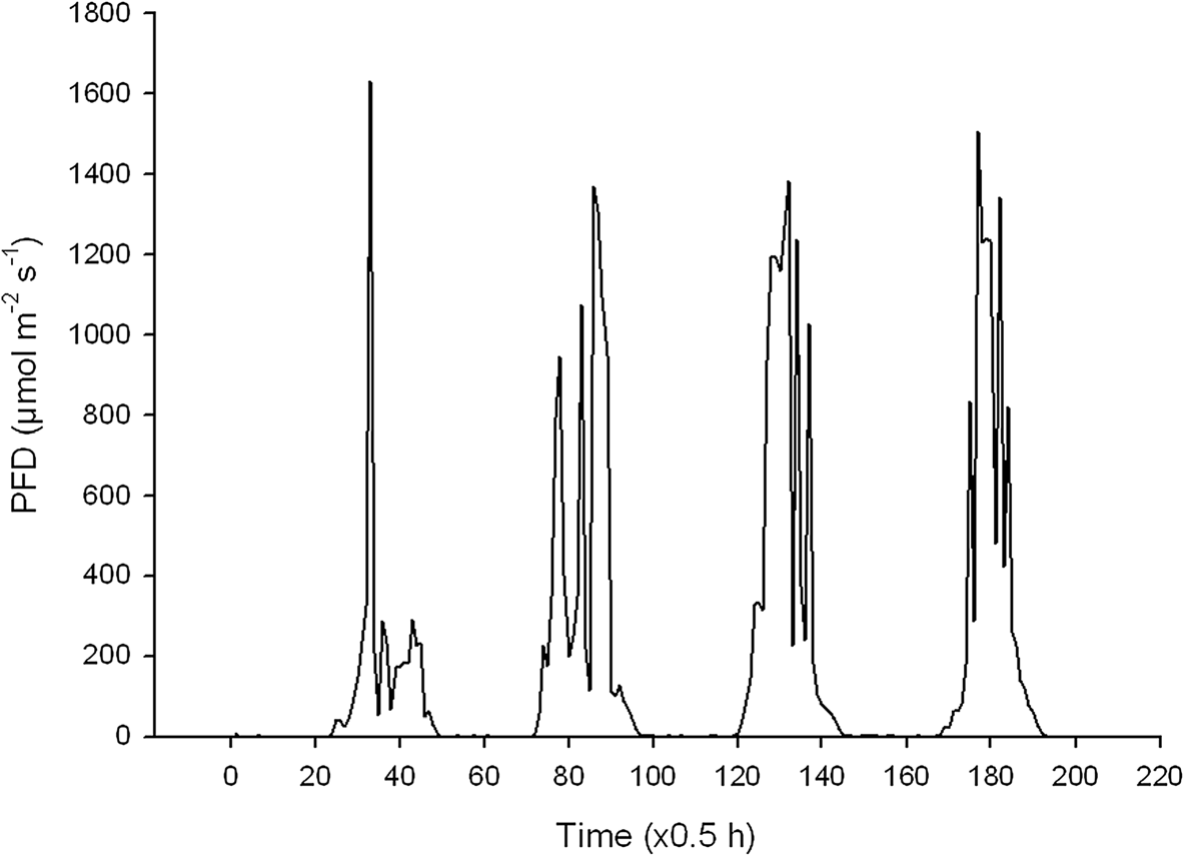
The photon flux density (PFD) of daylight during the experimental period.

**Figure 4:**
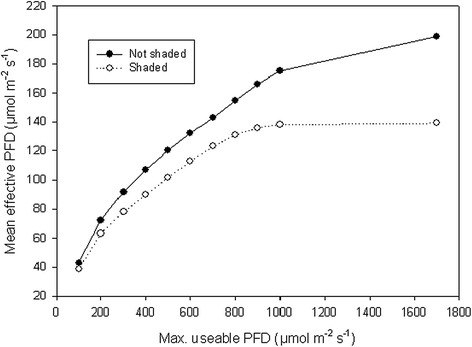
**Mean effective PFD at different threshold values of daylight useable for the growth of the microalgae.** PFD values above the threshold value were set to the threshold value, i.e. if PFD values above 300 μmol m^-2^ s^-1^ are recognised as having no effect, the effective PFD was set to 300 μmol m^-2^ s^-1^.

In this experiment the microalgae were grown outdoors during four days under the different CO_2_ treatments in full daylight and in 70% daylight by shading with white plastic (Figure 3). The bottles were closely placed adjacent to each other in water baths in rows with six bottles facing to the south. In the forefront row the culture received full daylight while the shade was given on the back row placed about 30 cm behind. Two water baths were needed for the 24 bottles including four CO_2_ and two light treatments. The temperature was as a mean 24°C, varying from a peak of around 30°C at midday down to around 22°C during the night. The experiment was carried out in mid-September and the day length was 12.5 h (06.50 – 19.30 h). The building of the power station was located a few meters north of the experiment. The PFD varied from 0 to a maximum of about 1600 μmol m^-2^ s^-1^ in full daylight and up to about 1100 μmol m^-2^ s^-1^ in shaded conditions (Figure 2). The mean PAR was 17.1 and 12.0 mol m^-2^ day^-1^ in full daylight and in shaded conditions, respectively. At the Meterological station 5 km from the experimental site (Østad, Sarpsborg, 59°N, 11°E) the corresponding daylight was measured to 19.7 mol m^-2^ day^-1^ when converted from global radiation to PAR (www.bioforsk.no, Agricultural Meteorological service). The higher measured value here was probably due to the light sensor with 180° view (Kipp & Zonen, CM11 pyranometer) and more diffuse light from the north since the building shaded for the light from this direction in the experiment. Mean effective PFD in the experiment was calculated by assuming that PFD above different threshold values (100, 200 μmol m^-2^ s^-1^ etc.) has no effect on the growth (has reached the light saturation level) of the algae (Figure 4).

The dry weight was measured by vacuum filtering 10 or 20 ml of culture through a 90 mm filter (Whatman GF/B, cat. No. 1821-090) and drying it in an oven for four hours at 100°C. No pore size of this filter is given, however, all algal cells remained on the filter since no colouration of the filtered water was observed. The data were analysed using the SAS-GLM procedure (SAS institute Inc., Cary, USA) based on the bottles as replicates (n=3).

## Results

### Experiment 1

From an initial concentration of 0. 2 g l^-1^, the dry weight reached its maximum level after three days at the highest PFD, since no further increase was found on the fifth day (Table 2). At low PFD, the algae concentration continued to grow until the fifth day. The dry weight production during the first three days was significantly lower in the 11.4% CO_2_ flue gas treatment (about 20%) than in the 6.7% and 2.5% CO_2_ flue gas treatments at 300 μmol m^-2^ s^-1^ PFD, while the decrease was less (5-10%) in the low PFD treatment. The dry weights at the two lower flue gas concentrations were slightly higher compared with the 2.6% control CO_2_ treatment using pure liquid gas. The dry weight production per day was about four times higher at 300 than at 75 μmol m^-2^ s^-1^ PFD, reaching about 70 g m^-2^ day^-1^. The dry weight produced per mol photosynthetic active photons was the same at both PFD levels. Increasing the flue gas concentration slightly decreased the O_2_ content and decreased the pH in the algae culture. Increasing the light level slightly increased the O_2_ content and decreased the pH.

**Table 2:** **The effect of different CO**_**2**_**concentrations supplied by flue gas (Fl) and one concentration supplied by pure liquid CO**_**2**_**gas (C) on pH, O**_**2**_**concentration in the culture and dry weight concentration (n=3, ±SE) after 3 and 5 days of** ***C. reinhardtii*** **grown at 75 and 300 μmol m**^**-2**^**s**^**-1**^**PFD**

		**Day 3**			**Day 5**		**Mean dry weight production**		
							**Day 0 - 3**		
**CO**_**2**_**treatment**	**PFD**	**pH**	**O**_**2**_**(mg l**^**-1**^**)**	**Dry w. (mg l**^**-1**^**)**	**pH**	**Dry w. (mg l**^**-1**^**)**	**mg l**^**-1**^**day**^**-1**^	**g m**^**-2**^**day**^**-1**^	**g mol**^**-1**^
2.6% C	75	7.1±0.0	7.5±0.2	798±27	6.9±0.1	1360±149	266±9	16.2±0.6	2.50±0.09
2.6% C	300	7.0±0.1	8.3±0.1	3297±46	6.8±0.1	3040±94	1099±15	67.0±0.9	2.59±0.04
2.5% Fl	75	7.3±0.1	7.8±0.4	877±84	7.1±0.1	1338±88	292±28	17.8±1.7	2.74±0.26
2.5% Fl	300	7.0±0.0	8.5±0.1	3527±48	6.8±0.1	3423±91	1175±16	71.7±0.9	2.77±0.04
6.7% Fl	75	7.0±0.1	7.0±0.3	845±36	6.6±0.1	1343±70	282±12	17.2±0.8	2.64±0.12
6.7% Fl	300	6.7±0.0	7.5±0.2	3663±124	6.4±0.2	3343±91	1221±41	74.5±2.5	2.89±0.10
11.4% Fl	75	6.8±0.1	6.4±0.2	777±14	6.5±0.1	1147±23	259±5	15.8±0.3	2.43±0.04
11.4% Fl	300	6.7±0.1	7.3±0.2	2933±36	6.5±0.1	2843±45	978±12	59.6±0.7	2.30±0.03
F-value and significance level:									
CO_2_		64.5***	50.9***	17.6***	25.6***	3.54*	17.6***	17.6***	5.73**
PFD		91.1***	77.4***	3512***	10.7**	403***	3511***	3511***	0.51
CO_2_ x PFD		3.79*	0.59	10.9***	1.32	1.25	10.9***	10.9***	0.89

The productivity during the first three days was calculated as the increase in culture concentration, in g dry weight production per m^2^ and day, as well as in g dry weight produced per mol of photosynthetic active radiation. F-values and significance levels are stated as follows: *p<0.05; **p<0.01; ***p<0.001.

### Experiment 2

The dry weight concentration increased significantly from the fourth to the fifth day in this experiment when the algae were grown at 300 μmol m^-2^ s^-1^ PFD and 24±2°C (Table 3). The dry weight production was significantly lower (about 25%) at the highest flue gas concentration compared with the other treatments. The increase in algae concentration from 1.6 to 2.9 g l^-1^ from the fourth to the fifth day resulted in an algal production of around 80 g m^-2^ day^-1^ in the different treatments, except in the 11.4% CO_2_ flue gas treatment, where the production was around 60 g m^-2^ day^-1^. At the end of the experiment, the pH decreased from 6.8 to 6.0 when the flue gas concentration was increased from the lowest to the highest level.

**Table 3:** **The effect of different CO**_**2**_**concentrations supplied by flue gas (Fl) and one concentration supplied by pure liquid CO**_**2**_**gas (C) on pH and dry weight concentration (n=3, ±SE) after 4 and 5 days of** ***C. reinhardtii*** **grown at 300 μmol m**^**-2**^**s**^**-1**^**PFD**

	**Day 4**		**Day 5**		**Dry weight increase**		
	**pH**	**Dry w. (mg l**^**-1**^**)**	**pH**	**Dry w. (mg l**^**-1**^**)**	**(mg l**^**-1**^**day**^**-1**^**)**	**g m**^**-2**^**day**^**-1**^	**g mol**^**-1**^
2.6% C	7.1±0.1	1457±56	6.9±0.1	2810±141	1353±92	82.6±5.6	3.19±0.22
2.5% Fl	7.0±0.0	1622±21	6.8±0.1	2943±48	1321±51	80.6±3.1	3.11±0.12
6.7 Fl	6.5±0.1	1635±21	6.3±0.1	2917±100	1282±79	78.2±4.9	3.02±0.19
11.4% Fl	6.4±0.1	1377±42	6.0±0.3	2380±81	1003±54	61.2±3.3	2.36±0.13
F-value and significance level:							
CO_2_	49.8***	17.6***	20.4***	7.31*	5.24*	5.24*	5.24*

The productivity from day four to five was calculated as the increase in culture concentration, in g dry weight production per m^2^ and day, as well as in g dry weight produced per mol of photosynthetic active radiation.

For significance levels see Table 2 footnote.

### Experiment 3

Reducing daylight by 30% shade had no significant effect on the growth of the algae (Table 4). The dry weight production was 12-14 g m^-2^ day^-1^ as a mean during four days. In this experiment, the dry weight production was 10-20% higher in the 2.5% CO_2_ flue gas treatment than in the other treatments. The production per mol photons was increased 40-50% by 30% shading.

**Table 4:** **The effect of different CO**_**2**_**concentrations supplied by flue gas (Fl) and one concentration supplied by pure liquid CO**_**2**_**gas (C) on pH and dry weight concentration (n=3, ±SE) after four days of** ***C. reinhardtii*** **grown in daylight or 70% daylight (shaded)**

				**Dry weight increase**	
**CO**_**2**_**treatment**	**Light**	**pH**	**Dry w. (mg l**^**-1**^**)**	**g m**^**-2**^**day**^**-1**^	**g mol**^**-1**^
2.6% C	Shaded	7.19±0.1	815±45	11.6±0.6	1.00±0.06
2.6% C	Daylight	7.1±0.0	762±43	12.4±0.7	0.72±0.04
2.5% Fl	Shaded	7.1±0.1	960±18	14.3±0.7	1.23±0.06
2.5% Fl	Daylight	7.1±0.1	935±45	14.6±0.3	0.85±0.02
6.7% Fl	Shaded	6.8±0.1	867±83	11.9±0.7	1.03±0.06
6.7% Fl	Daylight	6.7±0.1	780±46	13.2±1.3	0.76±0.07
11.4% Fl	Shaded	6.5±0.1	792±32	12.1±0.6	1.05±0.05
11.4% Fl	Daylight	6.4±0.2	797±41	12.1±0.5	0.70±0.03
F-value and significance level:					
CO_2_		110***	5.14*	5.14*	5.31**
Light		3.00	1.49	1.49	79.9***
CO_2_ x Light		1.00	0.36	0.36	0.63

The mean productivity during the four days of the experimental period was calculated as g dry weight production per m^2^ and day and as g dry weight produced per mol of photosynthetic active radiation.

For significance levels see Table 2 footnote.

## Discussion

The undiluted flue gas containing 11.4% CO_2_ caused a decrease in the dry weight production compared with lower flue gas concentrations (2.5 and 6.7%). This was particularly the case when the dry weight production was very high (up to 70-80 g m^-2^ day^-1^), obtained at 300 μmol m^-2^ s^-1^ PFD continuously applied (25.9 mol m^-2^ day^-1^ PAR). In low-light conditions, (continuously 75 μmol m^-2^ s^-1^ PFD or 6.5 mol m^-2^ day^-1^ PAR) or in sunny daylight with a day length of 12.5 h (17.2 mol m^-2^ day^-1^ PAR) when the growth rate was much lower, less or no negative effect was found of the undiluted flue gas. The question was whether the negative effect was related to the high CO_2_ concentration itself or to the accompanying air pollutants. Separate measurements indicated that the dissolved CO_2_ concentration in the culture with undiluted flue gas might be about 400 mg l^-1^ as compared with about 150 mg l^-1^ in diluted flue gas with a concentration of 2.5% CO_2_. This is far below the saturating level of CO_2_ in water that is about 1500 mg l^-1^ at 23°C and 1200 mg l^-1^ at 33°C. The present pollutant levels of NO_x_ and SO_2_ below about 50 mg m^-3^ in the flue gas seldom seem to cause growth reduction in microalgae (Matsumoto et al. [1997]; Douskova et al. [2010]; van den Hende et al. [2012]; Farrelly et al. [2013]; Jiang et al. [2013]). Other flue gas compounds such as CO, HCl, HF and heavy metals such as Hg have received little attention so far (van den Hende et al. [2012]). Probably the concentrations in the present flue gas were so low that they would have no effect on the growth. However, microalgae possess very high metal uptake capacities and accumulation in the cells will therefore take place (de-Bashan and Bashan [2010]). High CO_2_ concentrations (18-19%) from pure liquid CO_2_ gas, however, have recently been found to decrease the dry weight production in the same *C. reinhardtii* strain (Mortensen and Gislerød [2014]). Fischer et al. ([2006]) showed that cells of the same species were more susceptible to high-light stress under high CO_2_ concentrations than under low concentrations. In the present study, however, the negative effect of the high concentrations seemed to be more related to a high growth rate than to high-light conditions. It can also be noted that the maximum dry weight concentration reached in the algae culture in the flue gas decreased to the same extent (in percentage) as the dry weight production, indicating higher respiration or lower photosynthetic activity in the algae. The negative effect of the 11.4% flue gas in the present experiment was in contrast to the stimulating effect of flue gas, probably due to lower O_2_ content, found in some studies on microalgae (Vance and Spalding [2005]; Douskova et al. [2009]; Kliphuis et al. [2011]). Growing *Chlorella sp.* at 2-20% CO_2_ (v/v) simulating flue gas from biogas gave the same effect as growing the algae in food grade CO_2_ at the same concentrations (Douskova et al. [2010]). The environmental conditions could play a role here, and they might also be the reason for the positive effect of the moderate flue gas concentration with 2.5% CO_2_ in the present experiment in daylight.

The production at low-level light 24 h day^-1^ (6.5 mol m^-2^ day^-1^ PAR) was at the same level (around 14 g m^-2^ da^-1^) as at about a three times higher PAR in daylight, which demonstrates the limitation of the algae as regards utilising the high irradiance level. The productivity in daylight was typical of outdoor production systems and the high productivity was typical of controlled environmental conditions in laboratories (Grobbelaar [2012]). The light use efficiency in the present study was found to be the same in the range 75-300 μmol m^-2^ s^-1^ PFD. If we assume that all daylight above 300 μmol m^-2^ s^-1^ PFD has a value of 300 μmol m^-2^ s^-1^, the mean PFD of the daylight will decrease from 199 to about 90 μmol m^-2^ s^-1^ or 7.8 mol m^-2^ day^-1^ PAR. This level is comparable to the low-light level with artificial light applied 24 h day^-1^. In addition to the constraint caused by light saturation, the presence of a dark period is known to decrease algae growth much more than would be expected from the reduction in PAR (Jacob-Lopez et al. [2009]). This means that long day lengths and lower maximum irradiance levels at high latitudes would be beneficial for algae production during the summer months. However, short days and low PAR during large parts of the year make the production of algae impractical in such locations. Growing *C. reinhardtii* with the aim of using it to produce hydrogen should be based on using daylight in combination with flue gas in order to ensure a positive energy balance (Lam et al. [2012]). However, large-scale systems that can utilise the high irradiance levels of daylight much better than today (Slegers et al. [2013]) are a prerequisite for future energy-efficient hydrogen production using microalgae. Flue gas is an important CO_2_ source. However, while care should be taken to ensure a CO_2_ concentration that is optimal, the presence of pollutants in the flue gas in today’s industrial emissions seems to be less of a problem in relation to the growth of the algae.

## Competing interests

The authors declare that they have no competing interests.

## References

[B1] Borkenstein CG Knoblechner J Frühwirth H Schagert M Cultivation of *Chlorella emersonii* with flue gas derived from cement plant J Appl Phycol 2011 23 131 135 10.1007/s10811-010-9551-5

[B2] de-Bashan LE Bashan Y Immobilized microalgae for removing pollutants. Revew of practical aspects Bioresour Technol 2010 101 1611 1627 10.1016/j.biortech.2009.09.043 19931451

[B3] Douskova I Doucha J Livansky K Machat J Novak P Umysova D Zachleder V Vitova M Simultaneous flue gas bioremediation and reduction of microalgal biomass production costs Appl Microbiol Biotechnol 2009 82 179 185 10.1007/s00253-008-1811-9 19096837

[B4] Douskova I Kastanek F Maleterova Y Kastanek P Doucha ZV Utilization of distillery stilage for energy generation and concurrent production of valuable microalgal biomass in the sequence: Biogas-cogeneration-microalge-products Energy Conversion Management 2010 51 606 611 10.1016/j.enconman.2009.11.008

[B5] Farrelly DJ Everard CD Fagan CC McDonnell KP Carbon sequestration and the role of biological carbon mitigation: a review Renew Sust Energ Rev 2013 21 712 727 10.1016/j.rser.2012.12.038

[B6] Fischer BB Wiesendanger M Eggen RIL Growth condition-dependent sensitivity, photodamage and stress response of *Chlamydomonas reinhardtii* exposed to high light conditions Plant Cell Physiol 2006 47 1135 1145 10.1093/pcp/pcj085 16857695

[B7] Geier SC Huyer S Praebst K Husmann M Walter C Buchholz R Outdoor cultivation of *Chlamydomonas reinhardtii* for photobiological hydrogen production J Appl Phycol 2012 24 319 327 10.1007/s10811-011-9729-5

[B8] Gorman DS Levine RP Cytochrome f and plastocyanin: their sequence in the photosynthetic electron transport chain of *Chlamydomonas reinhardtii* Proc Natl Acad Sci U S A 1965 54 6 1665 1669 10.1073/pnas.54.6.1665 4379719 PMC300531

[B9] Grobbelaar JU Microalgae mass culture: the constraints of scaling-up J Appl Phycol 2012 24 315 318 10.1007/s10811-011-9728-6

[B10] Stocker TF, Qin D, Plattner G-K, Tignor M, Allen SK, Boschung J, Nauels A, Xia Y, Bex V, Midgley PM Climate Change 2013: The Physical Science Basis. Contribution of Working Group I to the Fifth Assessment Report of the Intergovernmental Panel on Climate Change ᅟ 2013 Cambridge University Press, Cambridge, United Kingdom and New York, NY, USA www.climatechange2013.orgᅟ www.climatechange2013.org

[B11] Jacob-Lopez AJ Scoparo CHG Lacerda LMCF Franco TT Effect of light cycles (night/day) on CO_2_ fixation and biomass production by microalgae in photobioreactors Chem Eng Process 2009 48 306 310 10.1016/j.cep.2008.04.007

[B12] Jiang Y Zhang W Wang J Chen Y Shen S Liu T Utilization of simulated flue gas for cultivation of *Scenedesmus dimorphus* Bioresour Technol 2013 128 359 364 10.1016/j.biortech.2012.10.119 23201515

[B13] Jo HJ Lee DS Park JM Modeling and optimization of photosynthetic hydrogen gas Production by green alga Chlamydomonas reinhardtii in sulphur-deprived circumstance Biotechnol Prog 2006 22 431 437 10.1021/bp050258z 16599558

[B14] Kastanek F Sabata S Solcova O Maleterova Y Kastanek P Branyikova I Kuthan K Zachleder V In-field experimental verification of cultivation of microalgae *Chlorella* sp. using flue gas from cogeneration unit as a source of carbon dioxide Waste Management and Research 2010 28 Kastanek F, Sabata S, Solcova O, Maleterova Y, Kastanek P, Branyikova I, Kuthan K, Zachleder V 961 966 10.1177/0734242X10375866 20671004

[B15] Kliphuis AMJ Martens DE Janssen M Wijffels RH Effect of O_2_:CO_2_ ratio on the primary metabolism of *Chlamydomonas reinhardtii* Biotechnol And Bioengineering 2011 108 2390 2402 10.1002/bit.23194 21538341

[B16] Lam MK Lee KT Mohamed AR Current status and challenges on microalgae-based capture Int J Greenhouse Gas Contr 2012 10 456 469 10.1016/j.ijggc.2012.07.010

[B17] Matsumoto H Hamasaki A Sioji NIkuta Y Influence of CO_2_, SO_2_ and NO in flue gas on microalgae productivity J Chem Eng Japan 1997 30 620 624 10.1252/jcej.30.620

[B18] Mortensen LM Nitrogen oxides produced during CO2 enrichment III. Effects on tomato at different photon flux densities New Phytol 1986 104 653 660 10.1111/j.1469-8137.1986.tb00666.x 33873870

[B19] Mortensen LM Gislerød HR The growth of *Chlamydomonas reinhardtii* as influenced by high CO_2_ and low O_2_ in flue gas from a silicomanganese smelter J Appl Phycol 2014 ᅟ ᅟ (in press) 10.1007/s10811-014-0357-8 PMC4387248 25866444

[B20] Nguyen AV Toepel J Burgess S Uhmeyer A Bilfernez O Doebbe A Hankamer B Nixon P Wobbe L Kruse O Time-course global expression profiles of *Chlamydomonas reinhardtii* during photo-biological H_2_ production PLoS ONE 2011 6 12 e29364 doi:10.1371 10.1371/journal.pone.0029364 22242116 PMC3248568

[B21] Skjånes K Lindblad P Muller J BioCO_2_ – a multidisciplinary, biological approach using solar energy to capture CO_2_ while producing H_2_ and high value products Biomol Eng 2007 24 405 413 10.1016/j.bioeng.2007.06.002 17662653

[B22] Slegers PM van Beveren PJM Wijffels RH van Straten G Boxtel AJB Scenario analysis of large scale algae production in tubular photobioreactors Appl Energy 2013 105 395 406 10.1016/j.apenergy.2012.12.068

[B23] Sueoka N Mitotic replication of deoxyribonucleic acid in Chlamydomonas reinhardii Proc Natl Acad Sci U S A 1960 46 83 91 10.1073/pnas.46.1.83 16590601 PMC285018

[B24] van den Hende S Vervaeren H Boon N Flue gas compounds and microalgae: (Bio-) chemical interactions leading to biotechnological opportunities Biotechn Advances 2012 30 1405 1424 10.1016/j.biotechadv.2012.02.015 22425735

[B25] Vance P Spalding MH Growth, photosynthesis, and gene expression in *Chlamydomonas* over a range of CO_2_ concentrations and CO_2_/O_2_ ratios: CO_2_ regulates multiple acclimation states Can J Botany 2005 83 796 809 10.1139/b05-064

